# Introduction, spread and selective breeding of crops: new archaeobotanical data from southern Italy in the early Middle Ages

**DOI:** 10.1007/s00334-024-00989-7

**Published:** 2024-03-08

**Authors:** Girolamo Fiorentino, Anna Maria Grasso, Milena Primavera

**Affiliations:** Laboratorio di Archeobotanica e Paleoecologia, Dipartimento di Beni Culturali, Università del Salento, Via D. Birago 64, 73100 Lecce, Italy

**Keywords:** Southern Italy, Early Middle Ages, *Linum usitatissimum*, *Vicia faba*, *Solanum melongena*, *Gossypium herbaceum/arboreum*

## Abstract

This paper presents a summary of the record of the cultivated plant macroremains from southern Italy during the early Middle Ages, with a focus on the recent discoveries of crop remains in Sicily. These have shed light on the introduction of new plants in the central Mediterranean region at ca. 500–1100 ce. Specifically, we are dealing with the first evidence of the adoption of two new varieties of crops, one of flax (*Linum usitatissimum* cf. conv. *mediterraneum*) and the other of broad (or faba) bean (*Vicia faba* cf. var. *equina*) during the 8th century, a period of Byzantine domination, and also the introduction of exotic plants such as *Solanum melongena* (aubergine) and *Gossypium herbaceum/arboreum* (cotton) following the Islamic conquest. In some cases these developments were to have long term impacts, for example in Sicily with regard to cotton, which became an important cash crop there in the 13th century, and throughout southern Italy with regard to a new variety of *Vicia faba* (*Vicia faba* cf. var. *major*), which was grown in Puglia from the late Middle Ages onwards.

**Supplementary Information:**

The online version contains supplementary material available at 10.1007/s00334-024-00989-7.

## Introduction

Few analyses of the plant macroremains identified from early medieval archaeological contexts in the south of Italy have been done (Grasso and Fiorentino 2009; Mercuri et al. 2015), so that regional studies have been presented only for certain regions such as Puglia (Grasso et al. 2018) and certain species such as *Olea europaea* (olive) and *Vitis vinifera* (grapevine) (Grasso 2016; Caracuta 2020). Regarding plant microremains, and pollen in particular, there are studies both on-site (Caramiello and Zeme 1994; Heim 1995; Vaccaro et al. 2015; Mercuri et al. 2019, 2020) and off-site (Di Rita and Magri 2009; Noti et al. 2009; Sadori et al. 2013; Mercuri et al. 2020; Michelangeli et al. 2022), enabling an understanding of the main palaeoclimatic and palaeoenvironmental variations at a subregional scale.

However, during the past few years, the available archaeobotanical dataset from southern Italy has been improved by two major archaeological projects: the ERC AdV-Grant project entitled “SIC-TRANSIT—The archaeology of regime change: Sicily in transition” (ID 693,600) and the PRIN project entitled “Byzantine Heritage of southern Italy” (ID 2017M93ABL). Both projects aim to assemble various knowledge bases with which to reconstruct the culture, economy, settlement patterns and landscape of southern Italy during the early Middle Ages, which had a fairly complex social and political structure. The initial Byzantine domination of the entire territory gave way to successive forms of interaction and variation in political and economic systems, sometimes happening in relatively brief periods. Indeed, in the southern part of Italy during the time span considered, frontiers moved back and forth as Byzantines, Muslims and Lombards fought for control until the definitive Norman conquest of the territory in the late 11th century. Based on an overview of the regional archaeobotanical evidence of macroremains of cultivated plants from Sicilia, Calabria, Basilicata and Puglia, the main goals of the present paper are: (1) to update the data to reflect the recent analyses of the two projects and (2) to highlight any diachronic changes in crop assemblages (through time) and to determine the existence and timing of any new crop introductions (Fuks et al. 2020), and changes in ways of farming. The new data and the exceptional state of preservation of some plant remains make it possible to contribute decisively to both the debate on Andrew Watson’s “Islamic green revolution” thesis (Watson 1974, 1983) and the more general theme of the spread of crops in the Mediterranean basin in the 1st millennium ce.

## Materials and methods

**Fig. 1:**
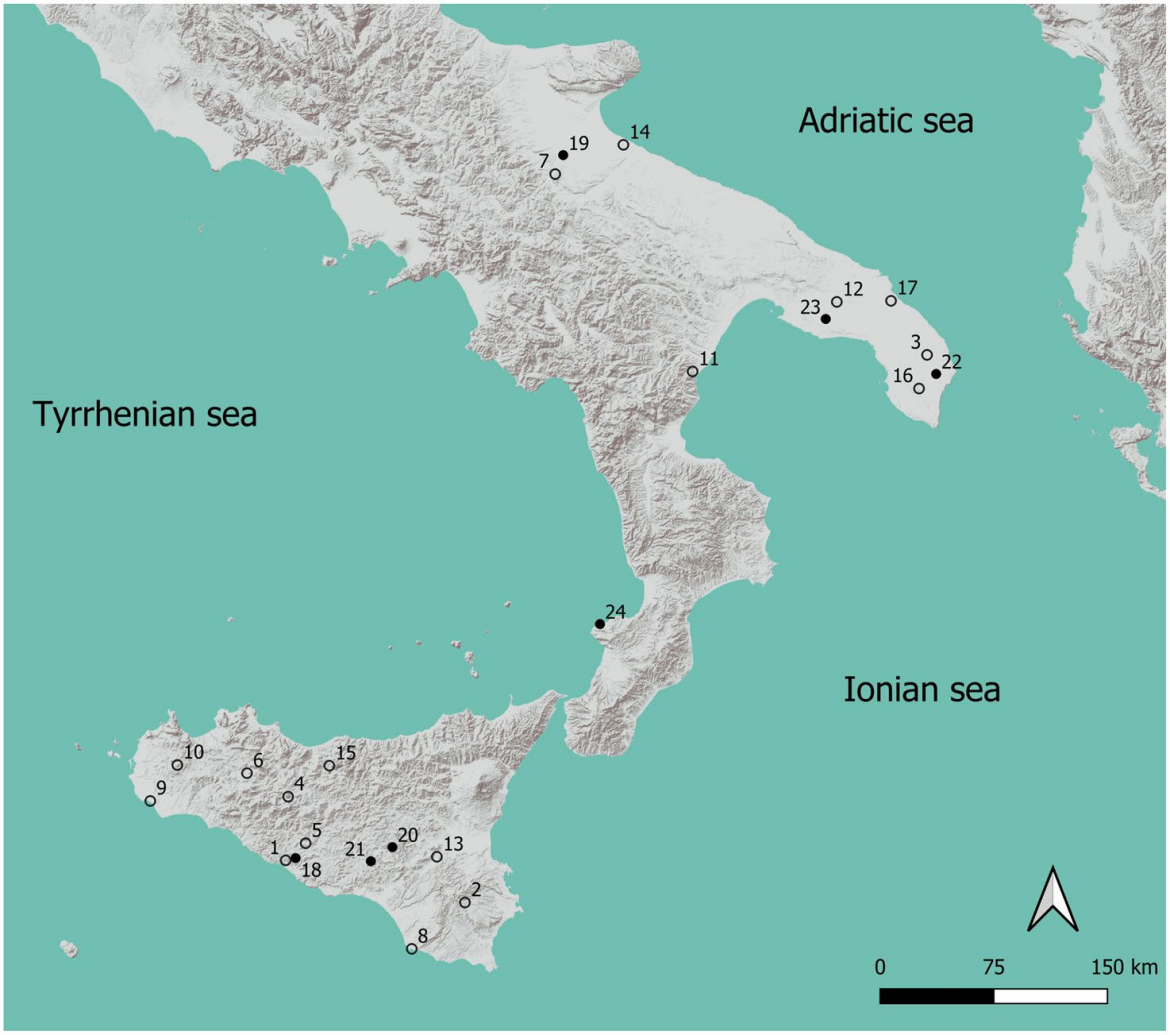
Map of southern Italy and Sicily showing the location of early medieval archaeological sites with archaeobotanical analysis. 1, *Valle dei Templi* Agrigento (AG); 2, *Akrai*, Palazzolo Acreide (SI); 3, *Apigliano*, Martano (LE); 4, *Casale San Pietro*, Castronovo (PA); 5, *Colmitella*, Racalmuto (AG); 6, *Contrada Castro*, Corleone (PA); 7, *Faragola*, Ascoli Satriano (FG); 8, *Kaukana* (RG); 9, *Mazara del Vallo* (TP); 10, *Monte Polizzo*, Salemi (TP); 11, *Murge di Santa Caterina*, Rocca Imperiale (CS); 12, Oria (BR); 13, *Rocchicella*, Mineo (CT); 14, *Salapia*, Cerignola (FG); 15, *Scillato*, Vallone Inferno (PA); 16, Supersano, *Scorpo district* (LE); 17, Valesio, Torchiarolo (BR). Open circles (○) = sites considered in the review with macroremains evidence 18, Favara, Contrada Saraceno (AG); 19, Herdonia, Ordona (FG); 20, *Piazza Armerina* (EN); 21, *Philosophiana*, Mazzarino (CL); 22, *Quattro. Macine*, Giuggianello (LE); 23, Sava, Camarda district, “*Paretone dei Greci*” (TA); 24, *Tropea* (VV); Black dots (•) = sites excluded from this review; references as in ESM 1

Archaeobotanical analyses were done on 25 archaeological sites in southern Italy dating from the 6th to the 11th century, and both published and unpublished archaeobotanical data are considered here (Fig. 1, ESM 1), but only the 17 sites with macrofossil crop remains are included in the dataset. Details of each of these sites are briefly described in ESM 2, with the name, location, chronology and relative cultural/political aspects, archaeological contexts sampled, number and volume in litres of sediment collected, recovery technique, total number and taphonomy of seed remains and references, including interim reports for Laboratorio di Archeobotanica e Paleoecologia (LAP), as well as student theses, even when unpublished. These show that while the methods used for collecting and processing samples are fairly standard, the type of archaeological contexts and related taphonomic processes are sometimes quite different: most remains are charred, but there are important exceptions such as mineralised and waterlogged remains, respectively from Mazara del Vallo and Supersano. There is large variation in sample size between the individual sites and correspondingly also the amount of plant remains recovered varies greatly. For data compilation and analysis, we excluded the assemblages from the Scillato site because these macroremains are reported only as presence/absence.

The 17 sites are grouped according to their chronology, presenting the absolute count for each taxon in terms of plant category (cereals, pulses, fruit and nuts, vegetables and textile plants) (Table 1). In the case of crop assemblages dated to several periods such as the 4th–6th centuries, the count is attributed to the later century. The nomenclature was updated in accordance with Zohary et al. (2012). For the cereals, the quantification represents only grains and no chaff remains, just as for grapes, of which only pips are counted and no other parts of the fruit. Regarding *Hordeum vulgare* (barley), the name refers to hulled forms, both 2 and 4–6 row types, while *H. vulgare* var. *nudum* (naked barley) is counted separately.

## Results

The total of 444 samples from 17 archaeological sites yielded 20,339 crop remains. The distribution of the results by century is given in Table 1. In general, cereals, fruit and nuts are the most abundant and widespread crops; legumes are always present but in lower numbers; vegetables are rare and found from only certain periods; herbaceous plants used for oil and/or fibre, mainly represented by *Linum usitatissimum* (flax), are frequent and abundant in specific contexts. Considering the various categories and the records of individual taxa in terms of date and grouping, it is possible to observe some trends.

**Table 1:**
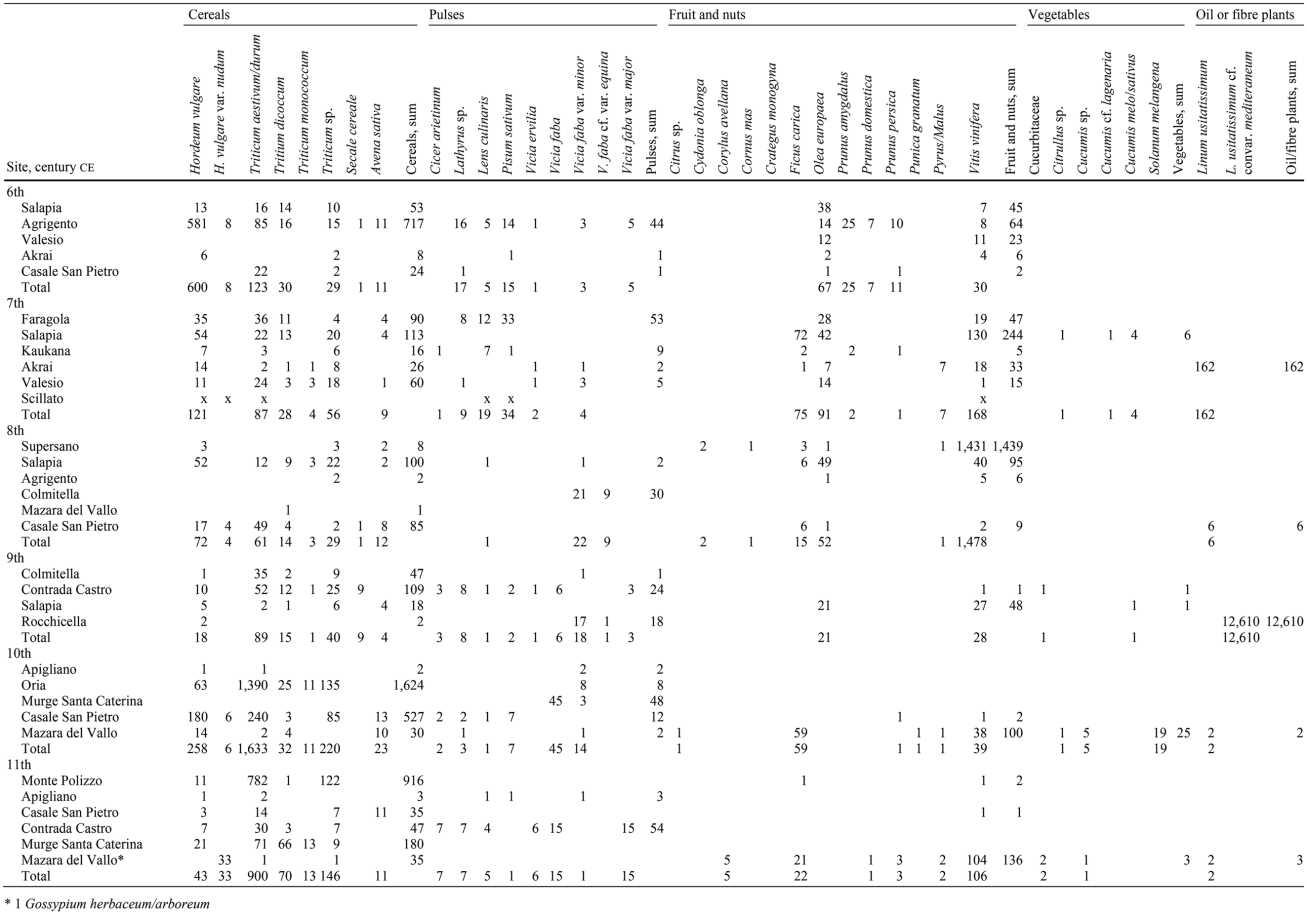
Crop assemblages from southern Italy and Sicily dating from the 6th to the 11th centuries ce

Absolute counts of taxa are given by site and century

**Fig. 2:**
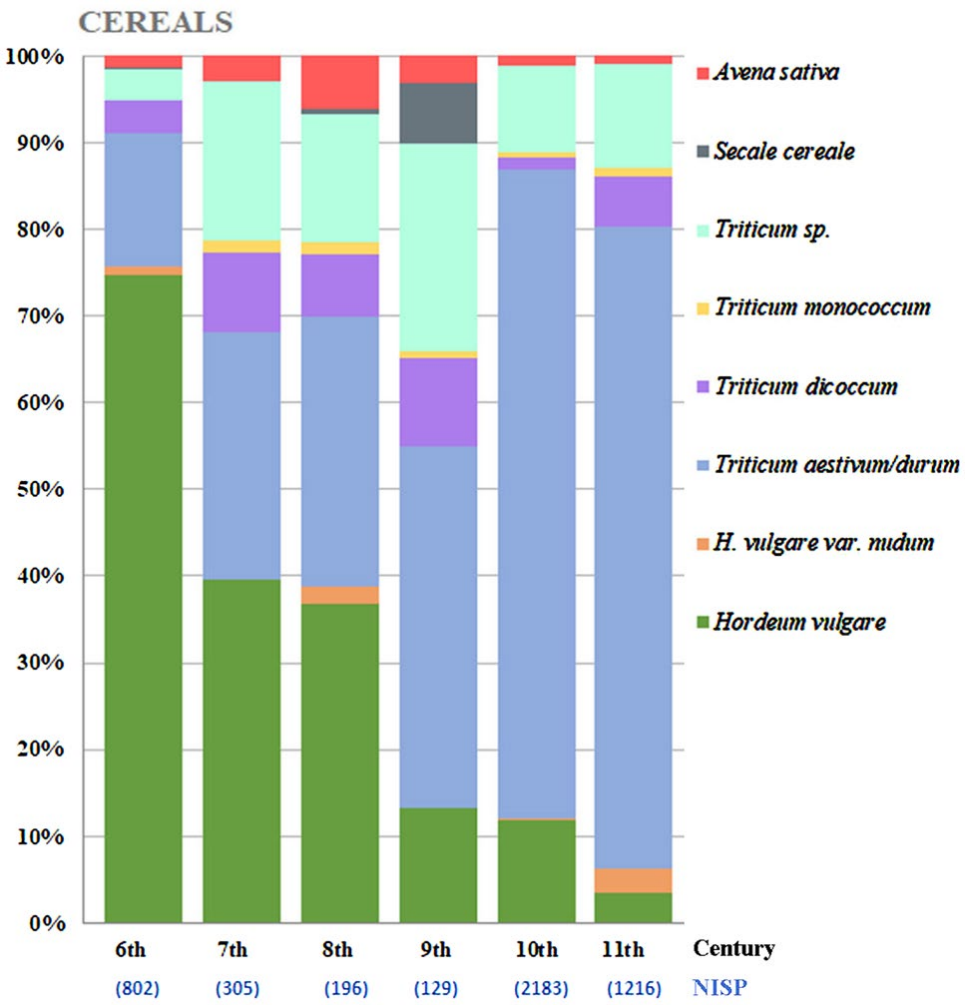
Percentage distribution of various cereal species for each period, from the 6th to the 11th century ce; absolute numbers of cereal remains are indicated in blue

*Cereals:* The period from the 6th to the 9th century sees a progressive move away from *Hordeum* (barley), initially the dominant cereal, towards *Triticum* (naked and hulled wheats), with small amounts of *Avena* (oats) and *Secale* (rye) (Fig. 2).

**Fig. 3:**
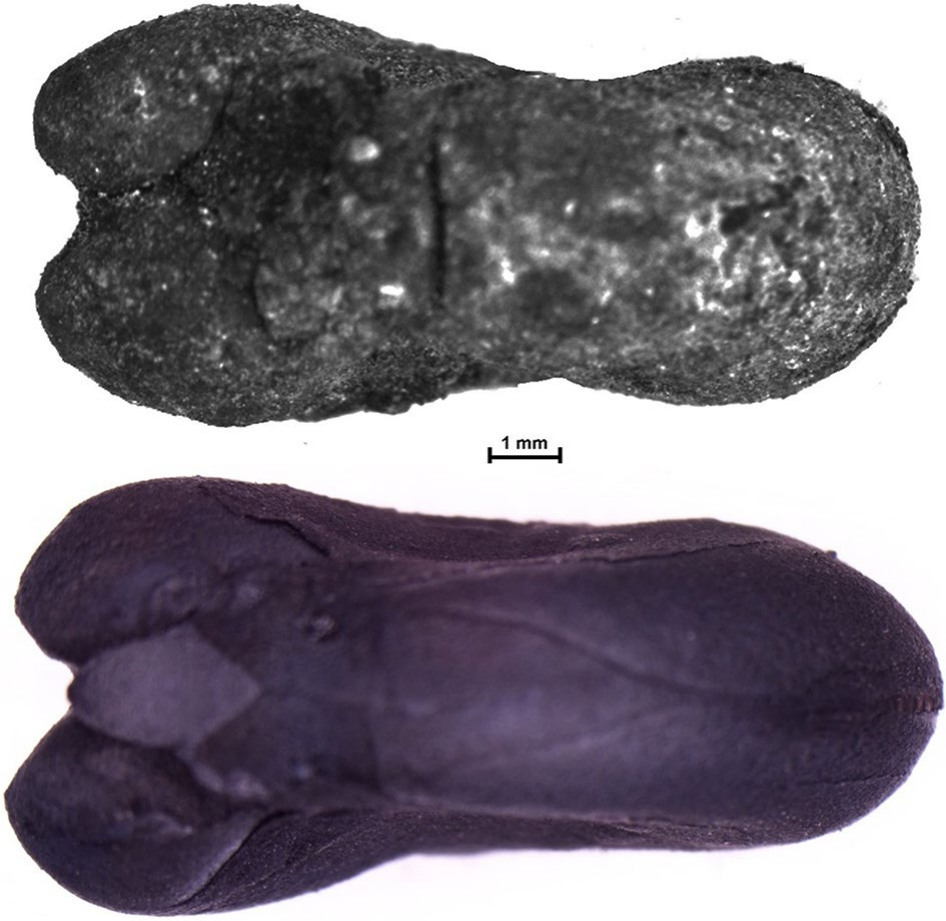
Above, charred *Vicia faba* bean from the archaeological context of Colmitella; below, charred modern *Vicia faba* var. *equina*, photographed in ventral view

*Legumes:* Despite being represented by only 319 remains in total, they are constantly present over time and always highly diversified in terms of taxonomic richness; the most interesting development is the appearance in Sicily in the 8th century of two new varieties of broad bean (Table 1). Currently, three main varieties of bean are commonly recognised, distinguished on the basis of the size of the seeds: the small-seeded field bean (*V. faba* var. *minor*), the medium-seeded horse bean (*V. faba* var. *equina*) and the large-seeded broad bean (*V. faba* var. *major*). Archaeobotanical studies demonstrate that, until Roman times, only a small-seeded variety was known (Hanelt 1972a, b; Schultze-Motel 1972). Together with *Vicia faba* var. *minor* (field bean), Grasso et al. (2020) recognized *Vicia faba* cf. *v*ar. *equina* (horse bean) in the Colmitella assemblage (Fig. 3; details in ESM 2 and 3). Furthermore, Stellati, Fiorentino (2016) and Castrorao Barba et al. (2021) identified *Vicia faba v*ar. *major*, but they did not describe the measurements of the beans.

*Fruit and nuts:* *Olea europaea* (olives), *Vitis vinifera* (grapes), *Ficus carica* (figs) and various species of *Prunus* (cherries, plums etc.) are the most abundant of these; other genera and species are represented by few remains but from several taxa, especially from the 6th, 10th and 11th centuries, periods from which the richer assemblages are from archaeological sites with waterlogged and mineralized preservation. Diachronically, grapes are the most ubiquitous fruit identified, while there is a relative drop in the presence of olives, which are absent from the assemblages of the last two centuries (Table 1).

**Fig. 4:**
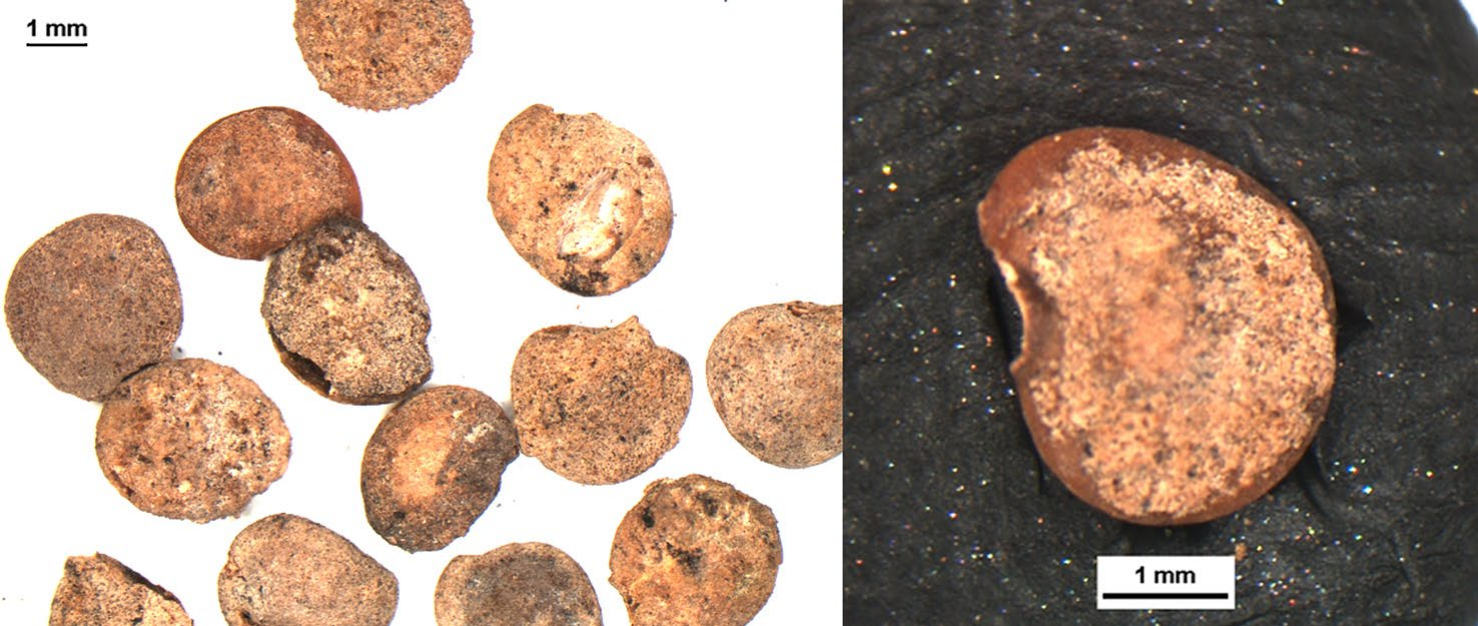
Mineralized seeds of *Solanum melongena* (aubergine) from Mazara del Vallo (Sicily)

*Vegetables:* Vegetable remains are rare and limited in number; unlikely to be found in archaeological layers, their preservation is usually seen only in specific contexts conducive to preservation by mineralisation, such as latrines or wells. In all centuries, the category is represented mostly by the Cucurbitaceae, *Cucumis* (cucumber or melon) and *Citrullus* (watermelon) (Table 1), although several mineralized *Solanum melongena* (eggplant) seeds were found from the 10th century Mazara del Vallo site in Sicily (Fig. 4; details in ESM 2 and 4).

**Fig. 5:**
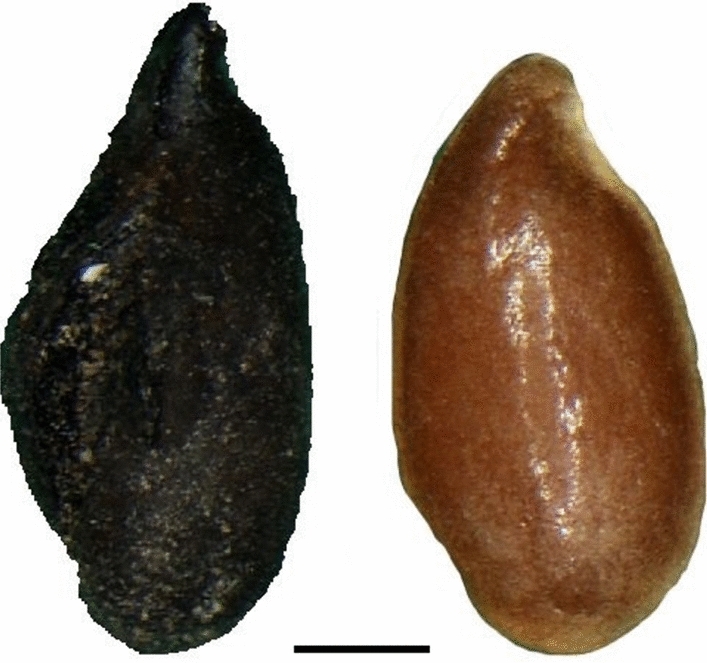
Left, charred archaeological *Linum usitatissimum* seed from Rocchicella; right, modern uncharred flax seed; scale = 1 mm

**Fig. 6:**
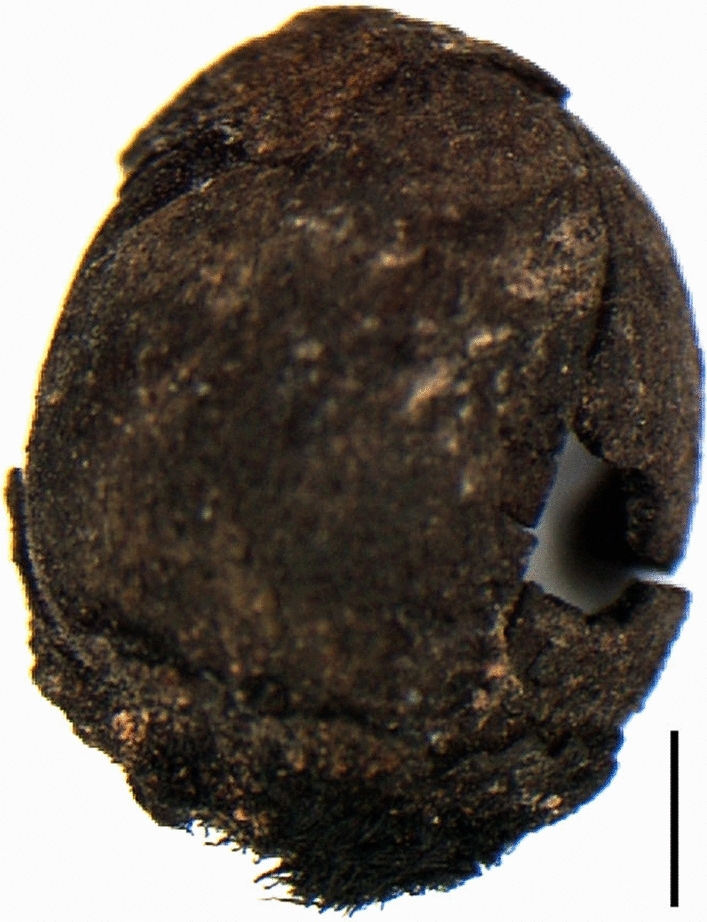
Charred seed of *Gossypium* sp. (cotton) from Mazara del Vallo (Sicily); scale = 1 mm

Among herbaceous plants grown for oil and/or fibre, the most widespread in southern Italy was *Linum usitatissimum* (flax) (Table 1); recent analyses seem to have recognized a new variety of flax in Sicily, from the 9th century Rocchicella site (Fig. 5, Grasso et al. 2021b; details in ESM 2 and 3), as well as the appearance of *Gossypium herbaceum/arboreum* (cotton) during the 11th century in Mazara del Vallo (Fig. 6; Primavera 2018; Fiorentino et al. 2021, details in ESM 2 and 4).

## Discussion

### General diachronic trends

The samples included in this analysis are distributed unevenly so that some regions, such as Calabria and Basilicata, are notably under-represented in the dataset. As a result, geographical patterns for these areas should be considered as having weaker support compared to those for other regions. Furthermore, the overall picture is affected by the specific nature of the contexts and taphonomic processes as in the case of the vegetables, which are under-represented because their preservation in sites like Mazara, Salapia and Monte Polizzo was by mineralization or, as is the case of Supersano, from continuous immersion in water for centuries. Lastly, there are taphonomic variations in the type of deposition bias the absolute counts, especially the 12,610 flax seeds found inside two pots from Rocchicella which amount to 62% of the total.

Anyway, considering the data now at our disposal, some general trends can be presented. The early medieval economy of southern Italy was largely based on cereals, but it seems that in the 9th century there was a change in the cereal taxa that were grown throughout the area being considered. For Sicily this phase corresponds to the beginning of the Islamic conquest of the island, while southern peninsular Italy remained under the control of the Byzantines and Lombards. On a regional scale, this trend, which involved an increase in free-threshing wheat at the expense of barley, was to continue over the 10th and 11th centuries, even as the political situation diversified, with the final Islamic conquest of Sicily. A recent high-resolution pollen record from south-eastern Sicily indicates an intensification of cereal growing during a phase of woodland reduction in the 10th–12th centuries (Michelangeli et al. 2022). This in turn might reflect a greater and more widespread cultivation of naked wheat at a time when the use of the landscape changed towards increasing cereal growing. The pollen data do not clarify which cereals were being grown, although where rachis remains are preserved in the cereal assemblages, we have evidence of the presence of *Triticum aestivum* (hexaploid bread wheat) and *T. durum* (tetraploid durum wheat). Cultivation of other cereals such as *Avena* and *Secale cereale* seems to have been less common, given that their remains were not found in large quantities, in addition to the fact that rye in particular was traditionally more widespread in northern medieval Italy (Rottoli 2014).

*Olea* and *Vitis* were the most common fruits in this part of the Mediterranean in the period under study. Analysing their records individually, what emerges in some centuries is that viticulture and olive growing seem to reflect contrasting agrarian and economic strategies. Vines seem to have been a constant presence, continuing to be cultivated in Sicily, as confirmed by the written sources (Branca 2003) and by the organic residue analyses of domestic containers (Lundy et al. 2021) and transport amphorae (Drieu et al. 2021), even after the Islamisation of customs and tastes might have reduced the consumption of wine. Concerning olives, the smaller number of finds of olive stones in the fruit assemblages from the 9th century and onwards suggests a drop in cultivation. In addition, organic residue analysis of Sicilian amphorae did not detect olive oil markers (Lundy et al. 2021). On a regional scale, pollen results from southeast Sicily show a reduction in olive growing following the Islamic conquest of the island after a period of growth in the 8th century (Michelangeli et al. 2022). This reduction seems to have lasted until the 12th century, when the Normans ruled. In contrast, a pollen sequence from southern Puglia indicates the widespread and continuous growing of olives there from the late Roman to the late medieval period (Di Rita and Magri 2009). This is confirmed by the charcoal data (Arthur et al. 2012; Grasso et al. 2018) and also by other archaeological evidence such as the introduction and spread of globular amphorae for bulk transport (Stranieri 2019), which could indicates an extension of olive cultivation and production from at least the 9th century, during the Middle Byzantine empire. There is a contrast between the data from pollen, charcoal and seeds etc. What is observed however might be explained by a change in agricultural strategy, with a probable increase of olive growing, but far from the settlements with on-site studies reported here, as the analysis of settlement patterns and land divisions in southern Puglia also seems to suggest (Stranieri 2019).

**Fig. 7:**
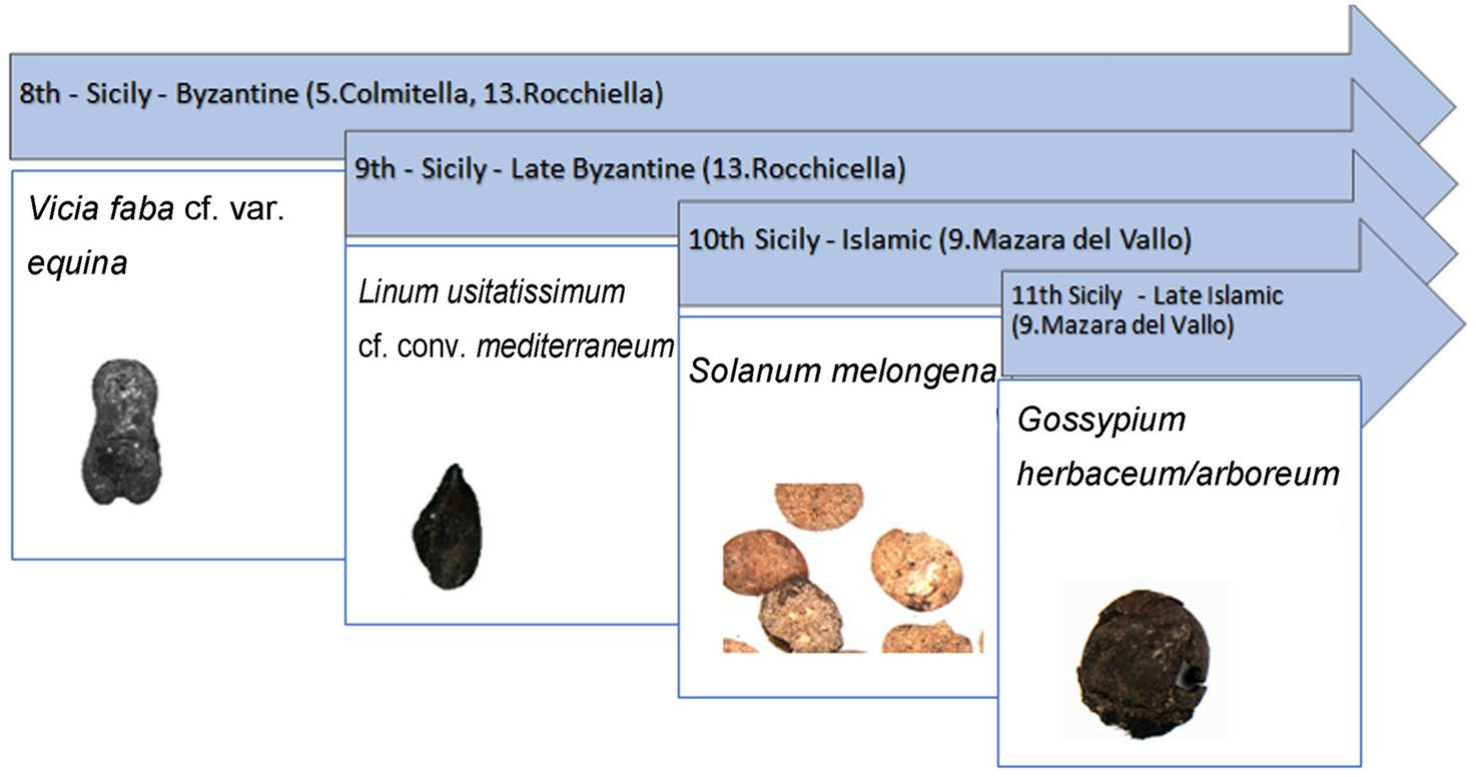
Dates of appearance of new crops during the early Middle Ages in the investigated area

Regarding the other main categories of crops, examination of the dataset indicated specific archaeological contexts with new introductions, both of new varieties of plants which were already grown in the region and also plants from elsewhere, which were already present in Sicily from 8th to 11th century contexts (Fig. 7). Examples of new varieties of plants already grown in the region are *Vicia faba* cf. var. *equina* and *Linum usitatissimum* cf. convar. *mediterraneum*, which were found from the Byzantine rural Sicilian settlements of Colmitella and Rocchicella respectively. More specifically, the Colmitella finds are dated to the late 7th or 8th century, which was a time of demographic and economic crises in Sicily as shown by a reduction in imports of pottery and amphorae (Rizzo 2023); in contrast, the Rocchicella finds are dated to the early 9th century, a period of greater stability of the rural settlements, indicated by the pottery assemblage, which includes a significant amount of imported transport amphorae (Arcifa 2023). The new varieties of crops are connected with various chronological and economic backgrounds and, without further information, it is not possible to understand whether they were introduced from somewhere else or were bred locally. The new plants which were brought from elsewhere, *Solanum melongena* (aubergine) and *Gossypium herbaceum/arboreum* were found in the secondmost important port of Sicily, Mazara del Vallo, from a time of true economic and demographic boom for Sicily in the 10th–11th century (Molinari 2015). In light of these considerations, it is intriguing to emphasize a significant aspect that arises from this analysis of the agricultural economy of southern Italy during the early Middle Ages. It suggests a difference between rural sites where new varieties were introduced, and urban environments where exotic plants were imported. These are further discussed, below.

### New crop varieties in the Byzantine period

The new varieties are of broad beans and flax. The identifications of *Vicia faba* cf. var. *equina* and *Linum usitatissimum* cf. convar. *mediterraneum* are probable but not certain (Grasso et al. 2020, 2021b), because the authors need to have fresh material from several gene banks for controlled charring experiments, in order to allow a better comparison of archaeological with recent material. Anyway, the comparison with archaeological material and other studies substantiates their hypothesis (ESM 3). Instead, regarding the *V. faba* var. *major* (broad bean) identified from the early medieval contexts of Agrigento and Corleone, it is not possible to say much, although we feel that the identification is problematic, since no measurements or other data have been published (ESM 3).

The two new crops, *Vicia faba* cf. var. *equina* and *Linum usitatissimum* cf. convar. *mediterraneum* were identified in the assemblages of Colmitella and Rocchicella respectively and dated to the late 7th and the first half of the 9th centuries, a period in which the Thema province, which included Sicily, Malta and Calabria, was firmly controlled by the Byzantine Empire, commercial contacts with North Africa were limited and Sicily played a key role in the main east-west route in the Mediterranean (Molinari 2009).

*Linum usitatissimum* and *Vicia faba* had long been present in southern Italy (Grasso et al. 2020, 2021b), as in the rest of the Mediterranean basin (Zohary et al. 2012; cf. Peña-Chocarro et al. 2019), so this suggests that the unusual finds of the Rocchicella flax seeds and Colmitella beans is not the result of taphonomy or sampling and recovery strategies. Moreover, analyses of the environmental and archaeological contexts and the archaeobotanical assemblage indicate that both were cultivated locally (Grasso et al. 2020, 2021b). Although it is currently not possible to understand whether the new varieties were introduced from elsewhere or were bred locally, it is possible to suggest some reasons why local farmers chose these new varieties. In the case of flax it is possible that the convariety *mediterraneum* was preferred because it has large seeds that make it suitable for the extraction of oil (Diederichsen 2019). In a period when there seem to have been variations in the economic system associated with olive growing in Sicily discussed above, linseed oil may have been a valid alternative, particularly for lamp oil. The use of linseed oil is believed to have been fairly common, at least in Sicily, given that the presence of a variety of flax suitable for oil is also known from Akrai between the 4th and 7th centuries, while an amphora, dated to the 10th or 11th century, which seems to have been used just for linseed oil, was identified from Mazara (Molinari and Meo 2021, p. 626).

In contrast, *Vicia faba* cf. var *equina*, may have been grown for a variety of reasons, one not excluding the other. In the first place it may have resulted from an attempt to improve crop yields, although it appears that the relationship between the size of the bean and the yield is not always direct (Cubero and Suso 1981; Agung and McDonald 1998; Al-Rifaee et al. 2004; Li and Yang 2014; Karkanis et al. 2018). A further aim may have been to reduce the content of secondary metabolites that make the beans indigestible (Zhang et al. 2022) or the substances that cause favism (Khazaei et al. 2019; Björnsdotter et al. 2021). Lastly, the greater height of the *equina* variety makes it easier to harvest the pods and results in a larger quantity of bean stalks, which can be used as fodder for livestock or even for non-food uses (Krenz et al. 2023).

The dating of both crops, which are the first finds from southern Italy, is reliable because the relative chronology based on material culture was confirmed by radiocarbon dating (ESM 3). The discovery of *V. faba* cf. var. *equina* from Colmitella is dated to the late 7th or 8th century, but the recent paper of Mir-Makhamad and Spengler (2023) has demonstrated that a large-seeded variety was clearly introduced in Tajikistan in the medieval period, before the Islamic conquests in the 7th and 8th centuries. The measurements of the beans, according to an ongoing study, seem to be comparable with var *equina*, but the debate about the earliest evidence of *equina* worldwide is complex and needs to be more fully investigated. Anyway, the *equina* variety was also found at Rocchicella in Sicily, from the late 8th or early 9th century ce (Grasso et al. 2020). Looking beyond the archaeobotanical evidence, it is possible that in this period, knowledge of a large-seeded variety of bean was by then widespread, as the expression *faba majores* (large beans) appears in the *Capitulare de Villis* (770–813 ce), a document about the governing of the royal estates of Charlemagne (Hanelt 1972a, b). Finds of *Vicia faba* var *equina* begin to appear in a wider area by the 9th century, as shown by a find from Suleimaniyah in eastern Iraqi Kurdistan (Helbæk 1963; Schultze-Motel 1972), becoming more numerous in the 12th century, as shown by the contexts of Lecce and Castro in southern Puglia (Grasso et al. 2020, 2021b) and that of Dury in north-eastern France (Bakels 1999), and from the 12th to the 14th centuries in Agios Mamas in Greece (Kroll 1999).

Regarding the *Vicia faba* var. *major* identified from the early medieval contexts of Agrigento and Corleone, it is not possible to say much, although we feel that the identification is problematic. Indeed, there is some agreement that in evolutionary terms, the *major* variety came later than *equina* (Cubero 1974; Nadal et al. 2003), but the earliest examples comparable to the *major* variety identified from southern Italy are considerably later, being dated to the late Middle Ages (D’Aquino et al. 2018; Grasso et al. 2021a).

There are currently no parallels for *Linum usitatissimum* cf. convar. *mediterraneum*, probably because archaeobotanists have carried out few systematic seed measurements on flax (cf. Karg et al. 2018), so it remains a challenge to establish where and when the different flax types were developed. However, genetic analyses conducted on modern varieties point to a Mediterranean centre of diversity and dissemination (Diederichsen 2019), which seem to be consistent with the results of Grasso et al. (2021b).

### Introduction of new exotic plants into islamic Sicily

Examining the introduction of new exotic plants into southern Italy during the Middle Ages, we cannot ignore the role of Sicily in the Islamic period from the 9th to the mid 11th centuries ce, especially considering the importance of the archaeobotanical assemblages recovered from the urban excavation of ancient Mâzar, now Mazara del Vallo (Primavera 2018; Fiorentino et al. 2021). This was the first city to be occupied by the Muslims in 827 ce; once included in the Dār al-Islām, it was part of an emirate, linked to Egypt and governed by the Kalbid dynasty, who lived in Balarm the capital of Sicily, now Palermo. Mâzar was the second most important port on the island after Balarm and is even cited in many documents of the Cairo Geniza (Goitein 1967–1993; Goldberg 2012).

In Mazara, the written sources can be cross-referenced with the plant record recovered from latrines, pits, wells, silos and occupation layers dated to the period from the 8th to the 13th century (ESM 4). The more than 3,000 plant macrofossils recovered there reflect a broad range of plants including cereals, legumes, vegetables, fruit and nuts, aromatic herbs and textile plants. Prominent among these are certain taxa included in the “exotic package” of 18 new plants that, according to Watson (1983), were introduced into the Mediterranean following the spread of Islam.

A number of seeds of *Solanum melongena* (Fig. 4) were found in the filling of Latrine 5 (ESM 4), from the Islamic phase, radiocarbon dated to 890–973 ce (95.4%, Hamilton 2021). The context of discovery and the date indicate the introduction to the urban diet of this plant of Indian origin in the late 10th century. These aubergine remains from Mazara are the most ancient evidence of the plant found so far in Italy and western Mediterranean Europe in general. The long distance trade of Mazar seem to have been with North Africa and to a lesser extent the Iberian Peninsula and the southern Tyrrhenian area (Molinari and Meo 2021). Also from Latrine 5 are numerous seeds of Cucurbitaceae (*Citrullus* and *Cucumis*), for which the mineralisation process was so advanced as to prevent precise identification of the species. Being perishable seasonal products that cannot travel long distances, they seem to indicate, like the aubergines, the consumption of fresh products such as watermelons, melons and cucumbers, which would have been cultivated locally, perhaps in vegetable gardens near the city.

The earliest evidence of *Gossypium herbaceum/arboreum* from Mazara del Vallo was also found in the Islamic levels (SU 78, ESM 4) (Fig. 6). The radiocarbon dating of a burnt seed was unsuccessful due to insufficient carbon content. This layer, corresponding to the filling of a small pit, yielded archaeological materials dated to the middle and the second half of the 11th century ce, the late Islamic phase. In this case too, we are dealing with the most ancient evidence of cotton from Italy and western Mediterranean Europe in general, although it has yet to be verified if it was locally grown, considering that at least for these chronological phases such remains are rare. In this regard, Andrew Watson cites various Arab sources including Ibn Hawqal, who claims that “*Cotton from Ddierba, Tunis and other parts of Ifriqiya were exported to Spain and Italy, where it was noted in Islamic times or after the Norman conquest near Catania, Agrigento, Mazara and Giattini*” (Watson 1983, p. 40, note 68). Other sources report that cotton was regularly grown from as early as the 10th century thanks to the skill and knowledge of the Sicilian peasants (Molinari 2021). From the archaeobotanical point of view, the question merits further study, in relation to both the species used (*Gossypium herbaceum* vs. *G. arboreum*), which would make it possible to trace both the specific routes by which cotton spread and was traded (Bouchaud et al. 2019) and its actual cultivation in Sicily. In any case, by the 13th century cotton was by far the most abundant taxon in the assemblages of Mazara: the record is rich, consisting of hundreds of charred seeds, whole and fragmented, recovered from the infill of two wells of the Schwabian phase (wells 1 and 2; ESM 4). Radiocarbon dating of a seed from this phase gave a date of 1,186–1,261 cal ce (95.45%, Hamilton 2021). The quantitative and chronological aspects of this discovery, together with the corresponding sources cited by Amari (1854–1872), which discuss the manufacture of cotton in Sicily in the 13th century, indicate that in these phases it was grown as a cash crop. These aspects can also be seen as evidence that the new crops introduced in the Islamic period were assimilated by the new political and economic systems and had a powerful impact on the economy and production processes of the island and beyond (Mazzaoui 1981). Indeed, during the late Middle Ages, in areas of cotton cultivation such as Sicily, Calabria and Puglia, the local weavers produced textiles for both for regional markets in the region and also for Venice and Genova in the north of Italy, and in some cases for export markets to Spain and France (Epstein 1989).

## Conclusions

The new data emerging from the recent research have made possible to expand the archaeobotanical database available for southern peninsular Italy and Sicily, for the 6th to the 11th centuries. This in turn has enabled preliminary identification of the diachronic trends of the archaeobotanical assemblages, showing a series of agricultural aspects that might be connected to the changes in political control of the area during this time. The Byzantines controlled large areas of southern Italy, including Sicily at first, and Lombard power was concentrated in Basilicata (ancient Lucania in southern Italy) and in the northern part of Calabria and Puglia there, while Islamic expansion mainly affected Sicily, but also certain cities of southern peninsular Italy for brief periods such as Bari and Taranto. Directly or indirectly, this political background may have played a role in the management of agricultural systems and innovation processes.

Olive growing seems to have been an important part of farming, given the evidence of its expansion everywhere in the early centuries of the Middle Ages, when the Byzantine empire was recovering from the Gothic War and the plagues, reorganising itself politically and economically, and dealing with its territorial contraction, especially in those areas of southern Italy that were affected by Islamic and Lombard domination. Further evidence of the political and economic reorganisation of Byzantine territories is provided by changes in the systems of farming, which seem to have favoured extensive olive growing outside the settlements.

Although the evidence for diachronic trends is still fragmentary and short of data, it shows complex dynamics between rural areas traditionally used for growing cereals, which were experimenting (Fuks et al. 2020) with new plant varieties, as shown by the discoveries *Vicia faba* cf. var. *equina* and *Linum usitatissimum* cf. conv. *mediterraneum*, and urban contexts, in which the dense Mediterranean trade network may have favoured the arrival of exotic crops such as cotton and aubergines and also the spread (probably with new cultivation systems) of other crops that had already been present previously such as *Citrus* and Cucurbitaceae. The introduction of new varieties and exotic crops during the Byzantine and Islamic periods is followed in subsequent periods by phases of diffusion of some of these plants, bringing significant innovations to the agricultural economy of southern Italy as a whole. In agriculture, innovation aims to increase production and improve quality, affecting various areas like crop varieties, growth conditions, tools and management practices. Success depends on local circumstances, including climate, economics, politics and culture. True innovation occurs when new elements are widely adopted due to general interest and demand (van der Veen 2010; Primavera 2018; Fuks et al. 2020). In fact, from a long-term perspective, some of these exotics or new plant varieties became well established in the following centuries. This process of introduction of taxa followed by their widespread diffusion brought changes to the ways of farming in the regions involved. For example, some studies on the period after the 11th century have indicated the widespread cultivation of cotton in Sicily (Amari 1854–1872; Mazzaoui 1981) and the growing of *Vicia faba* var. *major* in Puglia from the 12th to the 16th centuries (D’Aquino et al. 2018; Grasso et al. 2021a).

On the whole, our results show the importance of southern Italy and Sicily for developments in Mediterranean farming due to their role as areas where new farming techniques were tried and new crops introduced and probably then spread to the rest of the Mediterranean. In any case, this paper raises further questions and research themes such as the histories of broad/fava bean and flax which, in order to be addressed properly, require more archaeobotanical data and also to be cross-referenced with other sources of data.

## Supplementary Information

Below is the link to the electronic supplementary material.

Supplementary material 1 (DOCX 20.1 kb)

Supplementary material 2 (XLSX 19.3 kb)

Supplementary material 3 (DOCX 18.2 kb)

Supplementary material 4 (DOCX 796.7 kb)
