# Supplementary material for: Introduction, spread and selective breeding of crops: new archaeobotanical data from southern Italy in the early Middle Ages
Source: Veg Hist Archaeobot. 2024 Mar 8;35(1):117–28. doi: 10.1007/s00334-024-00989-7 (PMC12881126; doi:10.1007/s00334-024-00989-7)
Supplement: Supplementary file 1 — Supplementary material 1 (DOCX 20.1 kb) [file 334_2024_989_MOESM1_ESM.docx]

**Introduction, spread and selective breeding of crops: new archaeobotanical data from southern Italy in the early Middle Ages**

Girolamo Fiorentino, Anna Maria Grasso, Milena Primavera

**ESM 1**

**Sites mentioned in Fig. 1 and ESM 1 Site details with reference to archaeobotanical studies**

1. Agrigento

Stellati A, Fiorentino G (2016) Agrigento romana tra spazi naturali e spazi agricoli: il contributo dell’archeobotanica. In: Parello MC, Rizzo MS (eds), Paesaggi urbani tardoantichi. Casi a confronto. Edipuglia, Bari, pp 345- 352

Stellati A, Stella M, Della Penna V, Fiorentino G (2023) Lo studio archeobotanico dell'edificio termale rifunzionalizzato tra età tardoantica e altomedievale nell'insula IV. In Caminneci V, Parello MC, Pisciotta F, Rizzo MS (eds) Indagini archeologiche dell'Insula IV del Quartiere Ellenistico-Romano di Agrigento 2014-2018. ONE Group Edizioni: L’Aquila, pp. 491-509.

2. Akrai, Palazzolo Acreide (SI)

Stella M (2022) La dieta mediterranea tra archeologia, tradizione e innovazione: il ruolo dell’archeobotanica. Phd thesis, Università del Salento.

3. Apigliano, Martano (LE)

Fiorentino G (1999) Ricerche archeobotaniche e paleoambientali. In: Arthur P (ed) Da Apigliano a Martano. Tre anni di archeologia medievale (1997- 1999), Congedo Editore, Galatina, pp 54- 56.

Grasso AM, Fiorentino G (2009) L’ambiente vegetale: risultati delle nuove analisi archeobotaniche. In: Arthur P, Bruno B (eds) Apigliano: un villaggio bizantino e medievale in Terra d’Otranto. L’ambiente, il villaggio, la popolazione. Arti Grafiche Panico: Galatina, pp. 53- 56.

4. Casale San Pietro, Castronovo (PA)

Primavera M, Minervini I (2022) “Laudato ingentia rura, exiguum colito”. Assemblaggi archeobotanici a confronto nella Sicilia medievale: dagli orti di Mazara del Vallo (Trapani) ai campi aperti di Casale San Pietro (Castronovo di Sicilia, Palermo). In: Milanese M. (ed) IX Congresso Nazionale di Archeologia Medievale, Vol 2, Alghero 28 September-2 October 2022, All’insegna del Giglio, pp. 444-448.

Primavera M, Fiorentino G (in press) Le indagini archeobotaniche a Casale San Pietro (Castronovo di Sicilia-PA): agricoltura e ambiente vegetale tra età imperiale e basso medioevo. In Carver M, Molinari A, Orecchioni P (eds) Castronovo di Sicilia Archaeological Investigations and research 2014-2021.

5. Colmitella, Racalmuto (AG)

Stellati A, unpublished data, Laboratorio di Archeobotanica e Paleoecologia- Università del Salento

Grasso AM, D’Aquino S, Vacca E, Fiorentino G (2020) Medioevo è innovazione: breve storia della fava (Vicia faba L.) alla luce dei nuovi dati archeobotanici. Archeologia Medievale 47: 49-59

6. Contrada Castro, Corleone (PA)

Castrorao Barba A, Speciale C, Miccichè R et al (2021) The Sicilian Countryside in the Early Middle Ages: Human–Environment Interactions at Contrada Castro, Environmental Archaeology: 1-16

7. Faragola, Ascoli Satriano (FG)

Caracuta V, Fiorentino F, Turchiano M, Volpe G (2012) Processi di formazione di due discariche altomedievali nel sito di Faragola. Il contributo dell'analisi archeobotanica. Post-Classical Archaeology 2: 225-246

8. Kaukana, Santa Croce Caemerina (RG)

Ramsay J, Wilson RJA (2012) Funerary Dining in Early Byzantine Sicily: archaeobotanical evidence from Kaukan, Meditarch 25: 81–93

9. Mazara del Vallo (TP)

Primavera M (2018) Introduzione di nuove piante e innovazioni agronomiche nella Sicilia medievale: il contributo dell’archeobotanica alla rivoluzione agricola araba di Andrew Watson, Archeologia Medievale, XVL: 439-444

Fiorentino G., Porta M., Primavera M., Sellitto A. (2021) Mazara tra innovazione e continuità: il contributo dell’archeobotanica alla ricostruzione dei paesaggi, dei sistemi agricoli e delle abitudini alimentari tra periodo bizantino ed età moderna. In: Molinari A., Meo A. (eds) Mazara/Māzar: nel ventre della città medievale (secoli VII-XV). Edizione critica degli scavi (1997) in via Tenente Gaspare Romano, All’Insegna del Giglio, Sesto Fiorentino, pp. 567-594

10. Monte Polizzo, Salemi, (TP)

Stika HP, Heiss AG, Zach B (2008) Plant remains from the early Iron Age in western Sicily: differences in subsistence strategies of Greek and Elymian sites, Veget Hist Archaeobot 17: 139- 148

11. Murge di Santa Caterina, Rocca Imperiale (CS)

Coscarella A, Fiorentino G (2019) Il sito fortificato medievale di Murge di Santa Caterina (Rocca Imperiale, CS): un approccio integrato di archeologia. In: Coscarella A (ed), Ricerche. Studi in onore di Giuseppe Roma – Collana del Dipartimento di studi umanistici. Sezione di Archeologia XVI. Università della Calabria, Cosenza, pp 52- 64

Grasso AM, D’Aquino S, Vacca E, Fiorentino G (2020) Medioevo è innovazione: breve storia della fava (Vicia faba L.) alla luce dei nuovi dati archeobotanici. Archeologia Medievale 47: 49-59

12. Oria (BR)

Cocchiaro A, Napolitano C, Caprino P, D’Oronzo C (2015) Oria nell’Altomedioevo: un impianto per la trasformazione dei cereali fra IX e X secolo. In: Arthur P, Leo Imperiale M (eds.) Atti del VII Congresso Nazionale di Archeologia Medievale (Lecce 9-12 settembre 2015), Edigilio, Firenze, pp 387- 392

13. Contrada Rocchicella, Mineo (CT)

Grasso AM, D’Aquino S, Vacca E, Fiorentino G (2020) Medioevo è innovazione: breve storia della fava (Vicia faba L.) alla luce dei nuovi dati archeobotanici. Archeologia Medievale 47: 49-59

Grasso AM, Stella M, Arcifa L, De Benedetto GE, Fiorentino G (2021) Le vie del lino nel Medioevo: nuovi dati dal contesto bizantino di Rocchicella di Mineo (CT). Archeologia Medievale 48: 353- 370

Stella M (2022) La dieta mediterranea tra archeologia, tradizione e innovazione: il ruolo dell’archeobotanica. Phd thesis, Università del Salento.

14. Salapia, Cerignola (FG)

Girolamo F, D’Aquino S, Della Penna V (2022) Dalla laguna all’entroterra: il contributo dell’archeobotanica alla ricostruzione dei paesaggi e dei sistemi agricoli a Salapia tra Tardo Antico e Alto Medioevo. In: De Venuto G, Goffredo R, Totten DM (eds) Salapia-Salpi 1 Scavi e ricerche 2013-2016. Edipuglia, Bari, pp. 573-587

Girolamo Fiorentino, Valeria della Penna, Silvia D’Aquino (in press) Paleoambiente e paesaggi, sistemi agricoli e alimentazione vegetale a Salapia-Salpi: continuità e cambiamento tra Tarda Antichità e Basso Medioevo.

15. Vallone Inferno, Scillato (PA)

Forgia V, Martín P, López-García V et al (2013) New data on Sicilian prehistoric and historic evolution in a mountain context, Vallone Inferno (Scillato, Italy). Comptes Rendus Palevol 12(2):115-126. https://doi.org/10.1016/j.crpv.2012.11.002.

16. Scorpo district, Supersano (LE)

Grasso AM (2011) Analisi archeobotaniche a Supersano (Le): una comunità autosufficiente? Post-Classical Archaeologies 1: 297- 308.

Grasso AM (2012) Archeologia e storia della vite e del vino nel Medioevo italiano: il contributo dell'archeobotanica e nuove metodologie di analisi integrate per la caratterizzazione varietale, applicate ai contesti archeologici della Puglia meridionale. Phd thesis, Università degli Studi di Siena

17. Valesio, Torchiarolo (BR)

Stella M, Fiorentino G (2022) Paleoambiente e pratiche agricole a Torchiarolo (Br) tra il II a.C. e il VII d.C. In: D'Auria C, D'Onghia P (eds) Valesio. Il metanodotto Interconnessione Tap tra ricerca archeologica e tutela del paesaggio, Edipuglia, Bari, pp 163- 169

18. Contrada Saraceno, Favara (AG)

D’Oronzo C, unpublished data, Laboratorio di Archeobotanica e Paleoecologia- Università del Salento

19. Herdonia, Ordona (FG)

Heim J (1995) Appendice 1-Il paesaggio vegetativo. In Mertens J (ed), Herdonia. Scoperta di una città. Edipuglia, Bari, pp 321- 323

20. Piazza Armerina (EN)

Montecchi MC (2009) Indagini archeopalinologiche e microantracologiche nell’insediamento medievale nell’area della Villa del Casale di Piazza Armerina (Enna), con dati pre- e post-medievali, Phd thesis, Università degli Studi di Ferrara.

Mercuri AM, Montecchi MC, Florenzano A, Rattighieri E, Torri P, Dallai D., Vaccaro E (2019) The Late Antique plant landscape in Sicily: Pollen from the agro-pastoral villa del Casale - Philosophiana system. Quaternary International 499: 24-34

21. Philosophiana, Mazzarino (CL)

Vaccaro E, Torre GF (2015) La produzione di ceramica a Philosophiana (Sicilia centrale) nella media età bizantina: metodi di indagine ed implicazioni economiche, Archeologia Medievale 42: 53-91

22. Quattro Macine, Giuggianello (LE)

Fiorentino G (1999) Ricerche archeobotaniche e paleoambientali. In: Arthur P (ed) Da Apigliano a Martano. Tre anni di archeologia medioevale (1997-1999). Mario Congedo Editore, Galatina, pp 54 – 56

Grasso AM, Unpublished data, Laboratorio di Archeobotanica e Paleoecologia- Università del Salento

23. Camarda district, “Paretone dei Greci”, Sava (TA)

Stranieri G, Fiorentino G, Grasso AM, Napolitano C (2009) Organizzazione e trasformazioni dei paesaggi agrari medievali nel Salento. Un approccio archeologico e archeobotanico allo studio di una delimitazione agraria in pietra a secco (Sava – Taranto), Archeologia Medievale XXXVI:259- 271

Grasso AM, Fiorentino G, Stranieri G (2012) Brick in the wall: an archaeobotanical approach to the analysis of drystone structures (Puglia – Italy), SAGUNTUM- extra 13: 209- 216

24. Tropea (VV)

Caramiello R, Zeme A (1994) Analisi palinologiche in terreni dallo scavo del cortile del palazzo vescovile di Tropea, Archeologia Medievale XXI: pp.368- 370
