# Supplementary material for: Introduction, spread and selective breeding of crops: new archaeobotanical data from southern Italy in the early Middle Ages
Source: Veg Hist Archaeobot. 2024 Mar 8;35(1):117–28. doi: 10.1007/s00334-024-00989-7 (PMC12881126; doi:10.1007/s00334-024-00989-7)
Supplement: Supplementary file 3 — Supplementary material 3 (DOCX 18.2 kb) [file 334_2024_989_MOESM3_ESM.docx]

**Introduction, spread and selective breeding of crops: new archaeobotanical data from southern Italy in the early Middle Ages**

Girolamo Fiorentino, Anna Maria Grasso, Milena Primavera

**ESM 3**

**Archaeological specimens of *Vicia faba* cf. var. *equina* and *Linum usitatissimum* cf. convar. *mediterraneum***

*Vicia faba* is divided into distinct groups based on seed size: the dry seeds ranging from small-seeded minor beans (length: 0.65- 1.25 cm, mean 0.95 cm) to medium-seeded equina beans (length: 1.25 cm- 1.65 cm, mean 1.45 cm) and the large-seeded major beans (length:1.88 cm-3.05 cm, mean 2.46 cm) (Muratova 1931). Caracuta et al. (2015) carried out an experiment on modern faba bean to estimate the variability of size due to charring and they found that the length decreased by ~17%.

With regard to faba beans discovered at Colmitella (Racalmuto, AG) the charred cotyledons show a sub-rectangular shape; 30 samples of *Vicia faba* were measured by Grasso et al. (2020) and they found that some of them to be larger (length: 0.79 cm- 1.36, mean 0.97 cm) than the sub-circular field bean seeds commonly attested in archaeological contexts (and also present here) (Zohary et al. 2012; cf. Grasso et al. 2020). Their context of provenance is dated to 676-770 cal CE (95.5 %), consistent with the archaeological evidence (Grasso et al. 2020). Grasso et al. (2020) compared them with biometric data found in the literature, showing that the Sicilian attestation is the first that may be attributed to the *equina* variety.

In Sicily, the sites of Agrigento and Contrada Castro (Corleone, PA) yielded *faba* cotyledons that have been attributed to the *major* variety (Stellati, Fiorentino 2016; Castrorao Barba et al. 2021). Their contexts of provenance are dated to the 6^th^ century for Agrigento and the late 8^th^- 9^th^, and 10^th^- 11^th^ centuries for Contrada Castro. These could represent the earliest evidence of *V. b*. var. *major*, but Stellati, Fiorentino 2016 and Castrorao Barba et al. 2021 do not report the measurements of the samples and do not discuss the data, since we have not been able to verify and compare them with the seed size range of *major* beans.

Concerning flax, numerous charred flax seeds were recently recovered from the Sicilian village site of Rocchicella di Mineo. They were found inside two locally produced ceramic containers, dated to the early 9^th^ century, discovered next to a storage space near the craft area (Grasso et al. 2021). A sample of flax seeds underwent radiocarbon dating, yielding a calibrated value of 771-896 CE (87.9%) (LTL21250 = 1190 ± 30 BP) (Grasso et al. 2021). 120 seeds from the Rocchicella samples are measured and they are of large dimensions (5.6 ± 0.6 mm long, 2.4 ± 0.33 mm wide), even when compared to later contexts in southern Italy (e.g., Castro, LE, dated to the 16^th^ century) (Grasso et al. 2021). Comparing the results from Rocchicella with the reference collection created by Karg et al. (2018), it can be hypothesised, albeit with caution given that it involves a comparison between charred and fresh material, that the seeds belong to *L. usitatissimum* convar. *mediterraneum* (fresh seeds are 5.35 ± 0.26 mm long, 2.8± 0.15 mm wide), whose varieties include the so-called “Lino grande” (big flax), which originated in southern Italy (Dillman 1953).

**References**

Caracuta V, Barzilai O, Khalaily H et al (2015) The onset of faba bean farming in the Southern Levant, Sci Rep 5, 14370. https://doi.org/10.1038/srep14370

Castrorao Barba A, Speciale C, Miccichè R et al (2021) The Sicilian Countryside in the Early Middle Ages: Human–Environment Interactions at Contrada Castro. Environmental Archaeology: 1-16

Dillman AC (1953) Classification of flax varieties, 1946. USDA Technical Bulletin, vol. 1054. United States Department of Agriculture

Karg S, Diederichsen A, Jeppson S (2018) Discussing flax domestication in Europe using biometric measurements on recent and archaeological flax seeds – a pilot study. In Siennicka M, Rahmstorf L, Ulanowska A (eds.) First Textiles. The beginning of textile manufacture in Europe and Mediterranean. Oxbow Books, Oxford, pp 31- 38

Grasso AM, D’Aquino S, Vacca E, Fiorentino G (2020) Medioevo è innovazione: breve storia della fava (*Vicia faba* L.) alla luce dei nuovi dati archeobotanici. Archeologia Medievale 47: 49-59

Grasso AM, Stella M, Arcifa L, De Benedetto GE, Fiorentino G (2021) Le vie del lino nel Medioevo: nuovi dati dal contesto bizantino di Rocchicella di Mineo (CT). Archeologia Medievale 48: 353- 370

Muratova VS (1931) Common beans (Vicia faba L.). Bull. Appl. Bot. Genet. Plant Breed. 50: 1-298

Stellati A, Fiorentino G (2016) Agrigento romana tra spazi naturali e spazi agricoli: il contributo dell’archeobotanica. In: Parello MC, Rizzo MS (eds), Paesaggi urbani tardoantichi. Casi a confronto. Edipuglia, Bari, pp345- 352

Zohary D, Hopf M, Weiss E (2012) Domestication of plants in the Old World: the origin and spread of domesticated plants in south-west Asia, Europe, and the Mediterranean Basin. Oxford University Press, Oxford
