# Supplementary material for: Introduction, spread and selective breeding of crops: new archaeobotanical data from southern Italy in the early Middle Ages
Source: Veg Hist Archaeobot. 2024 Mar 8;35(1):117–28. doi: 10.1007/s00334-024-00989-7 (PMC12881126; doi:10.1007/s00334-024-00989-7)
Supplement: Supplementary file 4 — Supplementary material 4 (DOCX 796.7 kb) [file 334_2024_989_MOESM4_ESM.docx]

**Introduction, spread and selective breeding of crops: new archaeobotanical data from southern Italy in the early Middle Ages**

Girolamo Fiorentino, Anna Maria Grasso, Milena Primavera

**ESM 4**

**Mazara del Vallo (Sicily)** -. In 1997, during works for the construction of a parking lot, bulldozers removed part of the medieval archaeological deposit and structures of the ancient city of Mazara del Vallo (fig. 1). The portions that remained undamaged, especially negative archaeological structures, were excavated by means of rescue archaeology and the sediments sampled for future investigations (Molinari and Meo 2021). The stratigraphies brought to light belong to a period from the Byzantine to the Aragonese epochs (7^th^-15^th^ centuries), although the most significant contexts fall within the Islamic (10^th^-11^th^ centuries) and Swabian (13^th^ century) phases. These include waste pits, wells and decommissioned latrines which over time became a receptacle for garbage collected from various activities. Latrines in particular have been identified in several areas and several phases of life of the city. Some of them, in particular Latrine n. 5 (10^th^-12^th^ centuries) and Latrine n. 6 (13^th^ century) constitute an important source of data because they contain direct evidence of food plants eaten by the inhabitants of the city during those phases, whose corresponding archaeological levels are well-preserved. The rescue excavation removed the upper part of the stratigraphy and erased the horizontal relationship, so it is not possible to establish whether the latrines belonged to private houses or to public areas such as markets. The archaeobotanical assemblages recovered from L6 and L5 – from which aubergine remains were collected, specifically from layer SU19 (10^th^ century) and layer SU5 (12^th^-13^th^ centuries) – highlight mineralisation processes which have allowed some seeds, fruits and other parts of the inflorescences to be preserved in an exceptional way (the percentage of mineralised remains varies from 76% to 100%). Indeed, mineralisation has substantially affected specific categories of remains, with the greatest impact in all phases seen in tree fruits and vegetables. Important archaeobotanical assemblages were also recovered from wells n. 1 and n. 2, dated to the 13^th^ century. The contents of these structures, from which huge amounts of charred cotton remains were recovered, derive from the waste of domestic and/or craft activities carried out in the port city during the Swabian phase (Fiorentino et al. 2021).


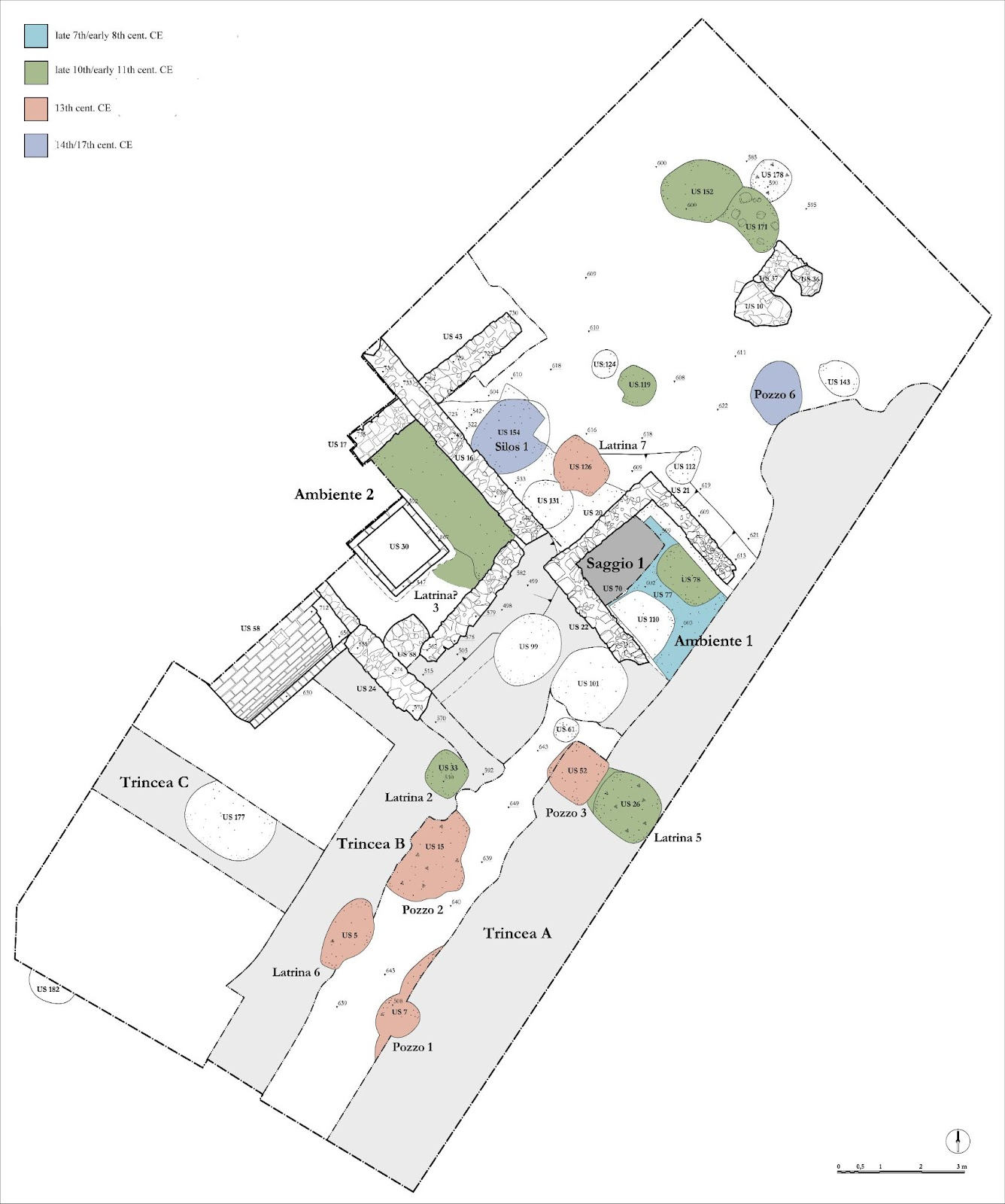


**Fig. 1** Plan of Mazara del Vallo, indicating contexts that have yielded archaeobotanical materials in the various periods (blue = late 7^th^-early 8^th^; green = late 10^th^-early 11^th^; pink = 13^th^; purple = 14^th^-17^th^ centuries CE)

**References**

Fiorentino G, Porta M, Primavera M, Sellitto A (2021) Mazara tra innovazione e continuità: il contributo dell’archeobotanica alla ricostruzione dei paesaggi, dei sistemi agricoli e delle abitudini alimentari tra periodo bizantino ed età moderna. In: Molinari A, Meo A (eds) Mazara/Mazar: nel ventre della città medievale (secoli VII-XV). Edizione degli scavi (1997) in via Tenente Gaspare Romano, Biblioteca di Archeologia Medievale, vol. 32. Edizioni del Giglio, Firenze, pp. 567-595

Molinari A, Meo A (2021) Mazara/Mazar: nel ventre della città medievale (secoli VII- XV). Edizione degli scavi (1997) in via Tenente Gaspare Romano. Edizioni del Giglio, Firenze.
